# The Effect of the Size of the Litter in Which the Sow Was Born on Her Lifetime Productivity

**DOI:** 10.3390/ani11061525

**Published:** 2021-05-24

**Authors:** Agnieszka Warda, Anna Rekiel, Tadeusz Blicharski, Martyna Batorska, Marcin Sońta, Justyna Więcek

**Affiliations:** 1 Polish Pig Breeders and Producers Association POLSUS, Ryżowa 90, 02-495 Warsaw, Poland; agnieszkawarda@polsus.pl (A.W.); blicharski@polsus.pl (T.B.); 2 Department of Animal Breeding, Institute of Animal Sciences, Warsaw University of Life Sciences—SGGW, Ciszewskiego 8, 02-786 Warsaw, Poland; anna_rekiel@sggw.edu.pl (A.R.); martyna_batorska@sggw.edu.pl (M.B.); justyna_wiecek@sggw.edu.pl (J.W.)

**Keywords:** sows, size of the litter of origin, reproductive performance

## Abstract

**Simple Summary:**

Improving reproductive traits, including increased fertility and prolificacy, is important for efficiency behind production, but difficult to achieve due to the low heritability for those traits, their dependence on the environment, as well as maintenance and nutrition. It is possible to achieve good results in reproduction using various methods of improving the characteristics of pig reproduction, such as breeding work, crossbreeding, selection programmes, optimisation of the rearing environment, and maternal effects. The litter of sow origin is one of the features worth using in practice, as it can have a significant impact on improving the fertility, prolificacy, and reproductive longevity of sows and is therefore a factor analysed in the work presented.

**Abstract:**

Improvement of lowly heritable traits is difficult, efforts must be made to take full advantage of the available information sources to improve them. The objective of the study was to determine the effect of the size of the litter in which the sow was born on her lifetime reproductive performance. Data on 22,683 litters were used to analyse the lifetime reproductive performance of 5623 Polish Large White sows. The sows from small litters (≤9) were on average the oldest at first farrowing, had the shortest herd life, the smallest number of litters, and the smallest sized litters (*p* ≤ 0.01). A positive relationship was established between the mean number of offspring born per litter and size of the litter in which the sow was born (*p* ≤ 0.01). For a sow to produce at least seven piglets per 100 days of reproduction, gilts from litters of at least 12 piglets should be selected for breeding.

## 1. Introduction

Genetically improving reproductive traits through traditional selection methods is small, as evidenced by the fact that the number of piglets born and reared to 21 days by Polish Large White (PLW) sows over 1963–2019 changed by about 2.0 and 2.2, respectively [1,2]. This is due to the long-term improvement of fattening and slaughter traits, which have negative genetic correlations with reproductive traits [3]. Selection for improved feed conversion has reduced fatness and increased muscling in pigs [4,5]. This was beneficial in terms of the carcass quality but had a negative effect on reproductive traits by decreasing fertility parameters, longevity, and lifetime production of the sows [6,7,8]. A slow but gradual improvement of reproductive traits in sows of maternal breeds was possible in Poland after the introduction in 2008 of a new assessment model accounting for reproductive traits. According to data from 2019, progress in the number of live piglets born per litter since the introduction of the BLUP model for sows PLW was +1.0 [2].

Breeding progress depends on evaluation accuracy, selection intensity, and heritability of the traits being improved. Since estimation of the coefficients of heritability (h^2^) for reproductive traits are low [3,9,10,11,12,13,14,15], it is difficult to improve them and the favourable changes are small. Reproductive traits are dependent on housing and feeding conditions [16], which further exacerbates the problem. Moreover, it was established that selection based on phenotypic traits does not imply that breeding progress is made. Therefore, in an effort to improve reproductive traits in pigs, other available sources of information, notably dam effects, began to be used [17,18,19,20].

Little information is available concerning the effect of dam on lifetime performance and reproductive longevity of the sows [20]. Most research has focused on the relationships between dam effects and the results of individual litters [19] rather than the sow’s lifetime litters. Jarczyk et al. [17] demonstrated that daughter fertility was positively affected by dam fertility, in particular by the weight of litter in which the dam was born. Inconsistent results were obtained in the studies of dam effects that accounted for the effect of size of the litter of origin and/or predominant sex of the litter in which the sow was born on reproductive traits [18,19,21,22,23,24,25], which suggests the need for further research. The analyses performed in the 1980s by Jarczyk and van der Steen [26] show that the best reproductive performance is achieved by the sows from litters of intermediate size (about 10.5), while the low birth weight of the piglets and high fertility of mothers reduces fertility in the daughters. However, according to Lewczuk and Rymkiewicz [21], breeding based on the gilts from small litters, which are characterized by better conformation and greater body weight, with low heritability of reproductive traits, may be the reason for low efficiency of selection for reproductive traits. Rekiel [24] and Ptak et al. [25] hold that gilts born in litters of 12–13 piglets should be selected as mothers for the next generation, while ensuring that high environmental, housing, and feeding standards are maintained [27].

The aim of the study was to determine the effect of the size of the litter in which the sow was born on her lifetime productivity.

## 2. Materials and Methods

Data on 5623 Polish Large White (PLW) sows from 160 herds in Poland were studied. Lifetime reproductive performance of the sows mated by pure-bred boars PLW was analysed from the date of first farrowing (2007–2009) to the date of removal from the herd. A total of 22,683 pure-bred litters were analysed (litters 1–15), the last farrowing was at 2017. Data were provided by the Polish Pig Breeders and Producers Association POLSUS. The animals with extreme values for the age at first farrowing (<250 and >550 days) or with missing parity were excluded.

Reproductive performance was analysed for the size of the litter in which the sow was born; age at first farrowing; age at culling; longevity (according to Engblom et al. [28], from the birth of first litter to culling from the herd); number of litters and piglets liveborn to sow during reproductive life; mean number of piglets liveborn per litter; and breeding herd efficiency, which show the number of piglets liveborn to the sow over 100 days of productive life.

Depending on the size of the litter in which the sow was born, data were divided into four groups: ≤9, 10–11, 12–13, and ≥14 piglets.

The reproductive performance was analysed using a mixed model, including the fixed effect of birth litter size (4 classes), and the random effects of herd and year of birth of the first litter. Differences were considered significant at *p* < 0.05. Multiple comparisons were made using Duncan’s test (IBM SPSS Statistics 27, 2021).

## 3. Results

The culling rate of the studied sows was stable in parities 1–3 (about 20%) and increased linearly afterwards (Table 1). After three litters were produced and reared, around 50% of the sows remained in the herd, which is consistent with the findings of Tarrés et al. [29], whereas after cycle 6 less than 20% of the studied sows remained in the herd. The culling rate varied according to the group. Forty-five percent of the sows remained in group I (≤9 piglets) after giving birth and rearing three litters, and around 8% more sows remained in groups III (12–13 piglets) and IV (≥14 piglets) (Table 2). A much greater difference in the culling rate of sows in various groups was observed after the first and second litter were reared. The culling rate of the sows from small litters compared to the sows from litters with at least 14 piglets, was higher after rearing the first and second litter by around 10 and 12%, respectively.

The sows from small litters (≤9 piglets) produced the first litter at a higher age and were culled the earliest from the herd (Table 3). Throughout their productive life, these sows gave birth to about 7% fewer piglets than the sows from the largest litters (at least 14 piglets) (group IV). The sows from litters of at least 12 piglets gave birth to 11 piglets in the first litter. The lowest herd breeding efficiency was observed in group I, and the highest in groups III and IV (*p* ≤ 0.01). Sows from the largest litters (≥14) had the longest reproductive life and produced the largest litters.

In our study, the proportion of sows that gave birth to more than 100 piglets during their herd productive lifetime was twice as high for sows from litters of at least 14 piglets (group IV) than for sows from the smallest litters (group I) (Table 4). Comparing the same groups showed that the proportion of sows that produced fewer than 30 piglets during their productive lifetime was 14% greater in group I than in group IV.

## 4. Discussion

Humpoliček et al. [18], Ptak [19], and Rekiel et al. [24] determined the effect of various dam effects on the reproductive performance of the sows. It appears that when improving reproductive traits, the size of the litter [19,23] and predominant sex of the litter in which the sow was born should be taken into account [22,23]. Ptak et al. [25] demonstrated that Polish Landrace sows from large litters (≥13 piglets) gave birth to and reared more piglets in the first four litters compared to the sows from litters with nine or fewer piglets. The authors believe that breeding work should account for the size of the litter in which the sow was born and choose next generation dams from litters of at least 12–13 piglets. Likewise, Rekiel et al. [24] concluded that next generation dams should be gilts born in litters of at least 13 piglets in the case of the PL breed and of 12 piglets for the PLW breed.

In the analysis of our results it is worth noting that sows born to dams from small litters (≤9 piglets) were the oldest at the start of reproductive life, and that they were the first to removal from the herd, which was responsible for their poorer performance during productive life. These results correspond with the data on female sexual and reproductive maturity and sow longevity and lifetime productivity. The onset of sexual maturity is influenced by both genotype and environment, notably feeding, health status, climate zone, season, and contact with a boar [30,31,32,33,34]. The age of sexual maturity is a moderately heritable trait (0.38), which means that it can be improved through selection [34]. Age at first farrowing is influenced by herd management, and reproductive efficiency is determined by the age of sexual maturity (age of first mating/insemination) of gilts [35], which is related not only to their body weight and body fat reserves [8], but also to the ovulation rate [36]. Reproductive maturity contributes to the sow’s reproductive potential in the herd, length of productive life, and lifetime productivity [30,37].

With respect to lifetime productivity, early puberty is highly beneficial [38]. It has been shown that younger gilts that reach sexual maturity before 185 days of age, produced more piglets over parities 1 to 3 when compared to older gilts [32]. Females with relatively late sexual maturity tend to increase the number of nonproductive days during their productive life. This undesirable phenomenon reduces farrowing frequency and lowers the economic efficiency of individual animals and the entire herd, as confirmed by the findings of Lucia et al. [39] and Tummaruk et al. [40]. Gilts that were introduced into the herd at an older age and served at >260 days of age have a shorter productive life than gilts integrated into the herd at a younger age [32]. In our study, first farrowing in groups II, III, and IV occurred 6, 5, and 11 days earlier than in group I, which means that puberty was attained by gilts that were younger about 1 week and originated from larger litters (≥10 piglets). Early reproduction had positive effects. Compared to sows from group I (which originated from the smallest litters and were introduced into the herd at an older age), sows from groups II, III, and IV had a longer herd life, their productive life increased by 6, 32, and 34 days, respectively, and they produced more litters throughout their productive life (by 0.1, 0.2, and 0.2, respectively). In our study, the mean productive life of the sows (from first farrowing to removal from the herd) was comparable or even longer than the results obtained in other experiments, which reported, for example, 617 days for Landrace sows (Sweden) [41], 602 days for Large White sows (Switzerland) [29], 579 days for commercial hybrid sows (Sweden) [28], and 489 days for Yorkshire sows (USA) [42]. Breeding herd efficiency in our study was also significantly (*p* ≤ 0.01) higher in groups III and IV than in groups I and II, which highlights the importance of the trait size of the litter in which the sow was born. The higher it was, the better the parameters of sow reproductive performance. It was calculated that for a sow to produce at least seven piglets per 100 days of reproduction, gilts from litters of at least 12 piglets should be selected for breeding. This is supported by the results of Ptak [19] and Rekiel et al. [24]. Kulisiewicz et al. [7] underlined that late puberty (late coming into first estrus), late mating of a gilt, and the resulting late farrowing is a seriously adverse factor and indicates a high risk of early removal from the herd, which is consistent with our study.

Longevity of females is a trait with a significant effect on swine farm profitability [42]. Efforts should be made to increase the productive herd life of sows since it reduces replacement costs, improves the health status, and helps increase the number of piglets reared [7]. Hoge and Bates [42], who studied longevity, showed that long lifetime production and low culling rates in pig herds bring significant economic benefits in the form of decreased sow replacement costs and a greater proportion of mature sows in a herd that have reached their maximum productivity. Sobczyńska et al. [20] concluded that with age, sows increase their reproductive capacity in successive cycles and therefore early removal from the herd is not economically justified. In our study, the culling rate of sows from the largest litters was considerably lower after rearing litters one, two, three, and four compared to the sows from small litters. Soltész et al. [43] have recognized that longevity should be treated comprehensively and so their study focused not only on the effect of genetic factors on this trait, but also on the management system in the reproductive sector, the culling system, the housing conditions, and the feeding program. A breeder, as reported by Engblom et al. [28], when deciding to cull a sow pays attention mainly to the number of litters produced, her reproductive status, health status, and herd structure. Although longevity of sows is crucial in terms of efficient and profitable pig breeding, around 50% of sows are annually removed from breeding herds [44]. The authors believe that a sow’s herd lifespan can be improved through genetic selection, but it is rarely accounted for in evaluation programs due to the long time intervals required to gather complete data on the productive period.

Hewitt and van Barneveld [45] estimated that a sow has a lifetime reproductive potential of 80–90 piglets, whereas Sobczyńska et al. [20] reported that the number of piglets reared per Polish sow of the maternal breed is only 30–40. According to Gill [46], a sow’s lifetime productivity of 30 to 40 piglets is normal and only few females produce and rear 60 piglets or more. A probable reason for such a low use of the sow’s reproductive potential is low realized fertility and/or a short reproductive life [20,46].

According to Lucia et al. [39], Stalder et al. [33,47], and Hewitt and van Barneveld [45], a sow should produce and rear at least three litters during her lifetime to make production marginally profitable, but the optimum number of farrowings has been reported around 5.5 [45]. Statistics show, however, that 40–50% of the sows are culled before parity 4 [29], which was also observed in our study. In each group, the mean number of litters produced by the sow was greater than 3, but this parameter characterized a large variation. In our study, we analysed the reproductive performance of the sows from parity 1 to 13 (group I) or 15 (groups II-IV). The observations of Rodriguez-Zas et al. [48] and Hoge and Bates [42] show that an excessive proportion of the sows are culled before peak productivity, with a mean number of litters lower than 5 (range of 3.1 to 4.6). Furthermore, almost one-third of herd females are removed as primiparous sows [49]. Segura-Correa et al. [50] underlines that removal of females from the herd not later than within several days after rearing the last litter, may reduce nonproductive days and increase farm profitability.

A sow’s production persistency and fertility in successive parities should be analysed together since the key to increasing the herd’s overall efficiency is to increase both the length of the sow’s herd productive life and the number of piglets reared per litter [33].

## 5. Conclusions

Based on the analysis of the lifetime reproductive performance of 5623 Polish Large White sows, it is concluded that:

- The sows bred from small litters were the eldest at first farrowing, as well as produced the smallest number of litters and the smallest sized litters;
- For a sow to produce at least seven piglets per 100 days of reproduction, gilts from litters of at least 12 piglets should be selected for breeding.

## Figures and Tables

**Table 1:** Number and percentage of sows, which have given birth to further parity.

Specification	Litter											
	1	2	3	4	5	9	7	8	9	10	11	12 and more
Sows Heads Percent	5623 100	4412 78.5	3555 63.2	2826 50.3	2140 38.1	1530 27.2	1055 18.8	682 12.1	414 7.4	249 4.4	118 2.1	79 1.4
Culling, % (Previous litter = 100%)	-	21.5	19.4	20.5	24.2	28.5	31.1	35.4	39.3	39.9	52.6	33.0

**Table 2:** Percentage of sows in each group, which have given birth to further parity.

Litter	Groups			
	I	II	III	IV
n	Size of the litter in which the sow was born			
	≤9 439	10–11 2143	12–13 2182	≥14 859
1	100	100	100	100
2	72.8	76.0	80.9	82.6
3	56.5	61.4	64.9	68.8
4	44.9	49.0	52.7	53.0
5	34.3	37.6	40.6	41.3
6	25.8	27.7	29.7	28.2
7	15.6	19.8	21.1	20.0
8	8.7	13.8	14.2	13.0
9	6.1	9.4	8.3	9.2
10	4.3	6.0	5.3	7.3
11	2.4	3.2	3.0	4.2
12 and more	1.4	1.6	1.1	2.0

**Table 3:** Size of the litter in which the sow was born and her reproductive performance.

Parameter	Groups				SE
	I	II	III	IV	
	Size of the Litter in Which the Sow Was Born				
	≤9	10–11	12–13	≥14	
Sows (*n*)	439	2143	2182	859	
Age at first farrowing (days)	357 ^ae^	353 ^acf^	352 ^b^	348 ^bd^	0.98
Age at culling (days)	1017	1019	1044	1041	11.36
Length of productive life (days)	660	666	692	694	11.37
Productive life:					
litters	3.8	3.9	4.0	4.0	0.07
piglets liveborn	42.9	44.4	46.4	46.4	0.83
Liveborn pigs per litter:					
first litter	10.8	10.8	11.0	11.0	0.04
mean for all litters	11.0 ^a^	11.0 ^a^	11.2 ^b^	11.3 ^b^	0.04
Breeding herd efficiency*	6.4 ^a^	6.6 ^a^	7.0 ^b^	7.0 ^b^	0.12

Means followed by different superscripts differ, ab, cd, *p* ≤ 0.01; ef, *p* ≤ 0.05. * Liveborn pigs per 100 days in production.

**Table 4:** Percentage of sows that produced a certain number of piglets during their productive lifetime.

No. of piglets	Groups			
	I	II	III	IV
	Size of the Litter in Which the Sow Was Born			
	≤9	10–11	12–13	≥14
<30	46.8	40.1	35.9	32.4
>60	18.9	21.1	27.1	23.9
including >100	5.2	9.2	8.8	10.9

## Data Availability

The data presented in this study are available on request from the corresponding author. The data are not publicly available to preserve privacy of the data.
